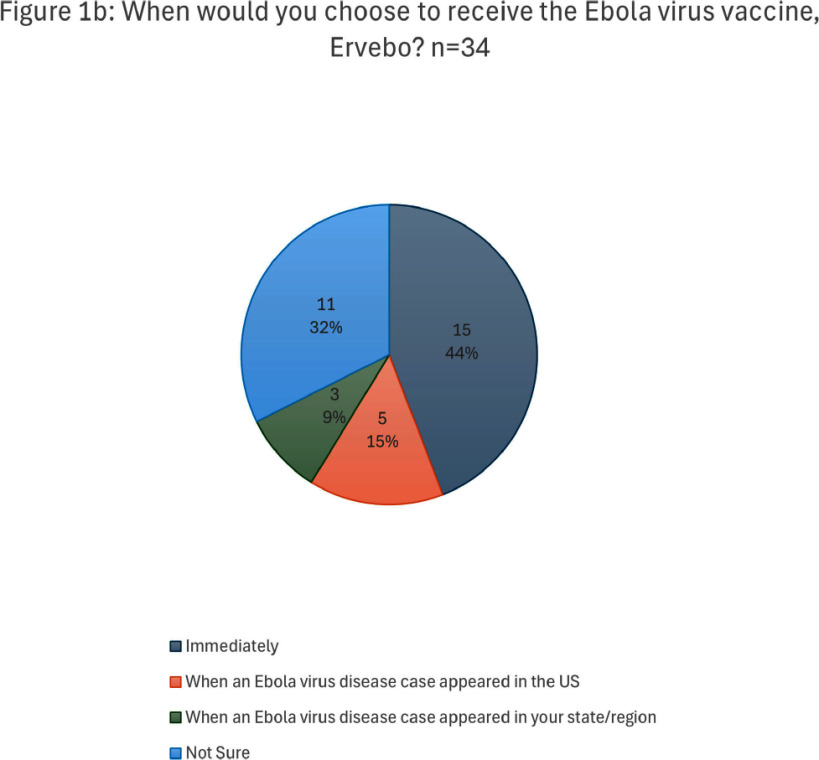# A Survey of Healthcare Worker Attitudes and Perceptions Toward the Ebola Vaccine, Ervebo

**DOI:** 10.1017/ash.2025.429

**Published:** 2025-09-24

**Authors:** Rinki Goswami, Anthony Lo Piccolo, Rachel Miller, Maria Frank, Corri Levine, Justin Chan

**Affiliations:** 1 New York University, Langone Health; 2 NYU Langone Health; 3 Denver Health & Hospital Authority; 4 University of Texas Medical Branch; 5 NYU Grossman School of Medicine

## Abstract

**Background:** Outbreaks of Zaire ebolavirus are an ongoing public health threat associated with high case fatality rates. The US Advisory Committee on Immunization Practices (ACIP) recommends preexposure vaccination with rVSV∆G-ZEBOV-GP Ebola vaccine (Brand name: Ervebo), which is effective in preventing disease caused by Zaire ebolavirus, to people at high risk for occupational exposure. We describe the perceptions and desire to be vaccinated with Ervebo among a subset of eligible US healthcare workers (HCWs). **Methods:** We conducted a cross-sectional online anonymous survey during March-October 2024, distributed to eligible HCWs at three Regional Emerging Special Pathogen Treatment Centers (RESPTCs): NYC Health + Hospitals/Bellevue, University of Texas Medical Branch, and Denver Health & Hospital Authority. **Results:** There were 66 responses (40% response rate), with the majority aged 30-49 years (63%), female (65%), and either a physician (42%) or nurse (27%). The majority (56%) had received some form of education on Ebola vaccines, most commonly through informational sheets or pamphlets (60%). Thirty-four (51%) were interested in (n=30) or already vaccinated with (n=4) Ervebo. Among those interested or already vaccinated, 44% would choose to receive the vaccine immediately, while 24% would get vaccinated if there were a case of Ebola virus disease (EVD) in the US. Among those not interested or unsure (n=32), most were concerned about risks of spreading the vaccine viral vector (44%), insufficient knowledge about the vaccine (31%), and unacceptable side effects (31%). Among all respondents, the most common concerns about adverse events included potential for a serious side effect (64%) and risk of arthritis (36%). Forty seven percent of respondents were concerned about the potential for spread of the vaccine virus vector. Respondents most frequently wanted more education on potential side effects (67%) and the risk of spreading the vaccine virus vector (59%). Among those not interested in vaccination or unsure (n=32), some may be convinced to accept vaccination if there were an EVD outbreak in the US (44%), if they better understood the risks and benefits of vaccination (34%), and if they better understood the vaccine safety (31%). **Conclusion:** During a period with no EVD outbreaks, a majority (51%) of eligible HCWs surveyed at three US RESPTCs were interested in or had received Ervebo. A significant proportion (24%) prefer to postpone vaccination until there is a case of EVD in the US. Deployment of Ervebo to eligible US HCWs may be optimized by addressing concerns identified in this study.

**Figure:**
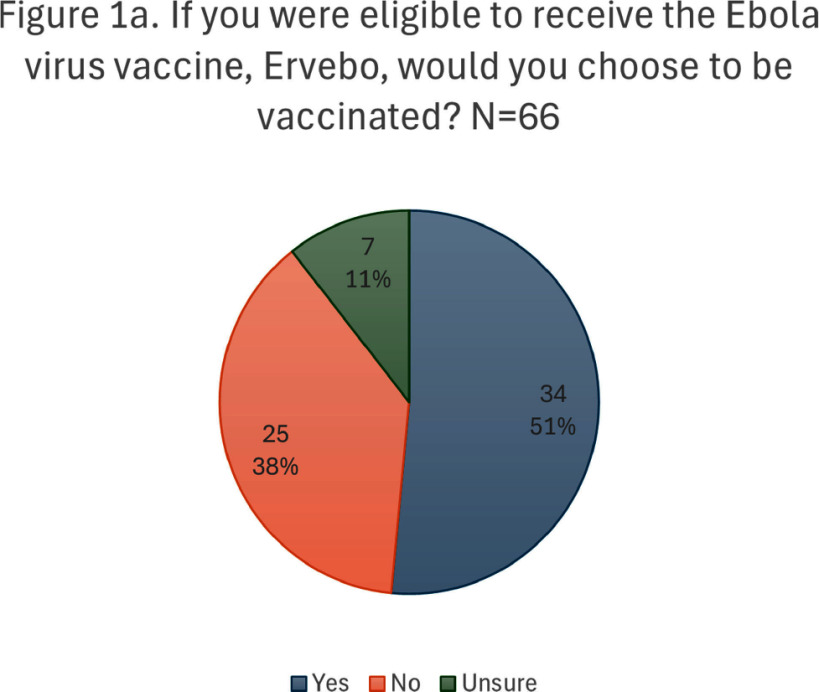

**Figure:**